# Perceived Importance of Climate Change Adaptation and Mitigation According to Social and Medical Factors Among Residents of Impacted Communities in the United States

**DOI:** 10.1089/heq.2019.0002

**Published:** 2019-04-11

**Authors:** Jennifer M. Kreslake

**Affiliations:** FHI 360, Washington, District of Columbia.

**Keywords:** climate change, public health practice, health equity

## Abstract

**Purpose:** To determine whether perceived importance of local climate change adaptation and mitigation efforts differs according to social or medical factors among residents of impacted communities.

**Methods:** An online survey was conducted among residents of California (Los Angeles/Orange), Florida (Miami-Dade/Broward), and Arizona (Maricopa) counties in July 2018 (*n*=605). Multivariable ordered logistic regression measured associations between the perceived importance of adaptation/mitigation approaches and income, race/ethnicity, and health conditions, controlling for age, political party, and county.

**Results:** Lower income was associated with higher perceived importance of improved emergency alerts, government-subsidized costs of household air conditioners and energy-efficient appliances, strengthening buildings against extreme weather, regulation of greenhouse gas emissions, urban planning using “cooling” technologies, and expanding community gardens/local agriculture. Black respondents perceived evacuation services for those with financial barriers during extreme weather, government-subsidized costs of energy-efficient appliances, and communication from government agencies about local climate impacts and mitigation as significantly more important compared to non-Black, non-Hispanic respondents. Hispanic respondents perceived significantly greater importance of improved emergency alerts and health care access during extreme weather, evacuation services for residents without transportation, government-subsidized costs of energy-efficient appliances, regulation of greenhouse gas emissions, communication from government agencies about local climate impacts and mitigation efforts, and intergovernmental cooperation on mitigation compared to non-Hispanic respondents.

**Conclusions:** Perceptions of the importance of specific local climate actions differ according to race/ethnicity and income. Community engagement is recommended to help local decisions reflect priorities of the most affected residents.

## Introduction

Communities across the United States are increasingly facing the disruptions from extreme weather and other environmental hazards long predicted by climate scientists.^1–7^ In addition to threatening local economies and infrastructure, these hazards place individuals with social or medical vulnerabilities at increased risk of death, injury, and exacerbation of symptoms of chronic health conditions.^1^ Low-income households, chronically ill individuals, and members of communities of color already affected by health disparities will face disproportionate burdens from these health impacts.^8^ Displacement due to extreme weather events, and the upheaval of social networks that can disrupt social support systems, further contribute to vulnerability to health impacts.^1^

Practitioners in emergency preparedness and disaster management use an all-hazards approach to prepare institutions for a variety of potential emergencies or disasters by strengthening systems and institutional capacities.^9^ As communities face mounting threats from extreme weather with increasing regularity, strengthening community resilience against extreme weather will require collaboration across numerous sectors and the deployment of multilevel interventions among individuals, social networks, institutions, and governments.^10^ To use terminology from the climate change field, “adaptation” interventions are those that help communities adjust to life under changing environmental conditions, whereas “mitigation” interventions aim to curtail the human activities (e.g., greenhouse gas emissions) that contribute to climate change.

Beyond managing immediate effects of extreme weather events, public health interventions must also address the upstream contributors to inequities (e.g., societal, economic, political) if they are to address health impacts in populations with greater social or medical vulnerability to climate change.^11^ Adaptation and mitigation approaches should be carefully implemented so they do not exacerbate health disparities.^12^ For example, an unintended consequence of increasing neighborhood green spaces may be gentrification, increasing property values to the point of displacing low-income residents and locally owned businesses. Using air conditioning during extreme heat waves—a short-term protective action—is costly, energy-intensive, increases street-level heat, and can overload power grids. Alternately, adaptation and mitigation can present opportunities to address systemic contributors to climate-related health disparities that have co-benefits for multiple public health issues.^12^

Americans who are disengaged from climate-related discourse are more likely to live in lower-income households.^13^ Local community engagement efforts are needed to gain input from these residents regarding their priorities and interests in community resilience efforts.^12^ More evidence is needed on whether commonly proposed adaptation and mitigation approaches address the expressed interests of low-income, chronically ill, and/or underrepresented groups. This study investigated whether residents with social or medical vulnerability in three U.S. regions recently affected by increasingly extreme weather and environmental hazards differ from other residents in their perceived importance of adaptation and mitigation approaches.

## Methods

All study activities received necessary IRB approvals.

### Sampling and recruitment

An online survey was conducted among residents of select counties from July 5–12, 2018. Participants were recruited from online research panels using a nonprobability sampling method. Potential respondents were randomly drawn from the population of panel members and invited to participate using email and mobile app alerts. Sampling quotas reflected regional distributions of age, gender, and race/ethnicity. Inclusion criteria were being aged 18 years or older, a current full-time resident of select counties in Southern California (Los Angeles, Orange, Ventura, or Santa Barbara), Florida (Miami-Dade or Broward), or Arizona (Maricopa) for at least 12 months, and recalling at least one of the following events within 50 miles of their home in the past 12 months: extreme heat (temperatures at least 10° higher than is normal for the region, lasting for several weeks), drought, flooding (flash floods, coastal or river flooding, and/or flooded city streets), hurricane/tropical storm, landslide/mudslide, and/or wildfire. Of the 1165 respondents who responded to the invitation, 77.4% met inclusion criteria. The survey had an 85.5% completion rate, resulting in a sample of 615 respondents. Of these, 2 were from Santa Barbara and 8 from Ventura counties; these counties were dropped from the analyses, resulting in a final sample size of 605.

### Variable construction

#### Dependent variables

Respondents were asked, “How important is it for your local government (city, county, or state) to do the following to help residents living in areas affected by extreme weather?” Respondents were presented with 31 health- or safety-related adaptation or mitigation measures in randomized order, rating the perceived importance of each using a bipolar Likert scale ranging from 1 (“not at all important”) to 5 (“extremely important”). Approaches were selected because they are recommended in the literature and/or being implemented in some U.S. communities.^14–17^ Table 2 includes the operationalized dependent variables. Measures encompassed emergency response, communication, support services, infrastructure, health care, structural approaches, approaches with co-benefits for public health, and intergovernmental cooperation. All distributions of dependent variables were highly left skewed (*p*<0.001 for each variable) and recoded into three-unit categorical measures for ordered logistic regression analyses.

**Table 2.** Perceived Importance of Public Health Adaptation (“A”) and Mitigation (“M”) Approaches

Type		Weighted mean (SE)	Weighted % rating “extremely important”
	**Emergency response**		
A	Restore power, water, or other utilities quickly during or after extreme weather	4.43 (0.05)	61.2
A	Provide emergency shelters during extreme weather	4.34 (0.05)	54.0
A	Improve communication to help emergency responders (e.g., ambulances, fire trucks) get to my neighborhood/building more easily	4.23 (0.05)	44.7
A	Improve evacuation routes to safer areas in extreme weather	4.14 (0.06)	44.2
A	Give neighborhood-specific information to the public about where to find help (e.g., maps and lists about types of help provided) during extreme weather	4.13 (0.06)	40.2
	**Communication**		
A	Improve emergency alert systems	4.32 (0.05)	52.6
A	Improve warning systems to alert the public when local high heat or poor air quality reaches dangerous levels	4.33 (0.05)	51.9
A	Improve warning systems to alert the public when contamination of local waterways, groundwater, or the ocean reaches dangerous levels	4.22 (0.06)	47.5
A	Quickly give me the information I need to take care of the health and safety of me and my family during extreme weather	4.22 (0.06)	46.9
M	Provide yearly updates about how extreme weather in my local area is changing over time	4.04 (0.06)	42.5
M	Give me information on how my community can prevent extreme weather from becoming stronger or more frequent in the future	3.96 (0.06)	38.1
A	Give me information on ways my community can keep extreme weather from damaging our area	3.95 (0.06)	37.6
	**Support services**		
A	Improve or create services to help people with disabilities, limited mobility, or the elderly in extreme weather	4.31 (0.05)	52.8
A	Provide transportation services to safer areas during extreme weather to people who don't have transportation	4.21 (0.06)	48.6
A	Improve or create services to help people who can't afford to evacuate in extreme weather	4.20 (0.05)	48.2
A	Give air conditioning units to people who can't afford them	3.99 (0.06)	39.9
A	Provide mental health services for people after extreme weather	3.90 (0.06)	35.5
	**Infrastructure**		
A	Improve drainage systems to prevent roads, businesses, and residential areas from flooding	4.26 (0.05)	47.4
A	Improve local infrastructure (e.g., roads, dams, levees, bridges) to be stronger during extreme weather	4.18 (0.06)	45.4
A	Require landlords and building owners to do construction or repairs that make housing safer during extreme weather	4.11 (0.06)	45.2
A	Improve or repair my neighborhood structures to prevent disruptions in power, water, or other utilities during or after extreme weather	4.16 (0.06)	44.3
	**Health care**		
A	Improve access to hospitals and health systems for health problems related to extreme weather	4.17 (0.05)	42.4
	**Structural approaches**		
A/M	Require public spaces and buildings to be designed to make cities cooler (e.g., install reflective roofs on buildings, plant rooftop gardens and trees on sidewalks, create parks and other green spaces)	4.13 (0.06)	44.0
M	Help cover the costs of energy-efficient appliances in homes of people who can't afford them	4.04 (0.06)	40.2
A/M	Provide loans, tax breaks, rebates, or giveaways to encourage residents to install roofs that use “green” technology to make cities cooler	3.98 (0.06)	39.9
M	Enact or enforce laws on businesses to lower pollution and greenhouse gas emissions in my area	3.99 (0.06)	38.7
M	Enact or enforce laws on households to lower pollution and greenhouse gas emissions in my area	3.91 (0.06)	37.4
	**Approaches with co-benefits**		
M	Restore natural open spaces and native wildlife or plant habitats	4.04 (0.05)	39.8
M	Improve transportation options to provide alternatives to cars (e.g., bike paths or trains)	4.03 (0.05)	39.4
M	Support and expand community gardens or local agriculture (e.g., farm stands and local farmers)	3.94 (0.06)	38.4
	**Intergovernmental cooperation**		
A	Work with other governments (city, state, national, or international) to develop recovery plans after extreme weather	4.12 (0.05)	42.1
M	Work with other governments (city, state, national, or international) to prevent extreme weather from getting worse	3.99 (0.06)	40.0

1=Not at all important; 5=extremely important; *n*=605.

#### Independent variables

Individual-level indicators of social and medical vulnerability to health impacts of climate change were classified as predictors.

##### Household income

Low-income households fell at or below 200% of the 2018 federal poverty level, calculated as a function of household size and income.^18^ A dichotomous measure (0=not low-income, 1=low-income) was constructed based on this calculation. A separate dichotomous variable for respondents from households at or below the 2018 federal poverty level was constructed for certain analyses.

##### Health status

A dichotomous variable was constructed indicating whether the respondent reported at least one chronic medical condition (asthma, chronic obstructive pulmonary disease, cystic fibrosis, diabetes, obesity, heart disease, or other lung diseases). Other dichotomous variables were created for pollen allergies, mental health conditions, and disabilities (including physical disability, blindness, or being deaf).

##### Race/ethnicity

A categorical measure was created to compare Black or Hispanic respondents to other respondents (1=white/Asian/other, non-Hispanic, 2=Black or African American non-Hispanic, 3=Hispanic).

#### Covariates

##### Age

A categorical measure of age group was used to control for age-related attitudinal differences.

##### Political party

The respondent's political party affiliation (0=Republican or Independent/Republican-leaning; 1=Democrat or Independent/Democrat-leaning) was used as a control variable based on established associations between political party and opinions about climate change.^13^

##### Geographic region

An indicator variable for county of residence was used to control for known and unknown influences of geographic variation on perceptions of the importance of interventions.

### Statistical analysis

Post-stratification sampling weights were constructed using county-level U.S. Census estimates for race, Hispanic ethnicity, household income, educational attainment, and age group.^19^ Multivariable ordered logistic regression analyses were conducted using Stata v.15.1 for each dependent variable. Multilevel mixed effects models were not used because of the small number of county- or state-level groups.^20^ Instead, an indicator variable was used for county as a covariate. Predictors were selected from independent variables that demonstrated statistically significant associations with dependent variables in bivariate analyses. Stepwise analyses and Wald tests were used to determine model fit. All models controlled for county, age, and political party affiliation (“other covariates”).

## Results

### Sample description

The sample was divided across counties in three metropolitan areas: Central Arizona (Maricopa County, *n*=205), Southern California (Los Angeles County, *n*=155 and Orange County, *n*=44), and Southeast Florida (Miami Dade County, *n*=98 and Broward County, *n*=103) (Table 1). Approximately 10% (10.9%) of the overall sample was Black or African American, and over one-third (38.4%) was Hispanic. Slightly less than one-third of respondents (30.4%) reported having one or more chronic health conditions, one-quarter (23.2%) had pollen allergies, and slightly more than 10% had a disability (12.6%) and/or a mental health condition (10.5%). Democrats (including Democrat-leaning Independents) comprised the majority of this sample (63.8%), while nearly one-third (32.8%) were Republicans (including Republican-leaning Independents).

**Table 1.** Sample Descriptives (Weighted %)

	Arizona	California		Florida		Total sample (*n*=605)
	Maricopa (*n*=205)	Los Angeles (*n*=155)	Orange (*n*=44)	Broward (*n*=103)	Miami-Dade (*n*=98)	
Age						
18–24	10.8	20.3	17.5	15.4	8.8	14.2
25–34	18.6	25.6	28.9	17.2	15.5	19.1
35–49	33.0	25.6	27.0	21.3	35.0	28.9
50–64	21.1	18.1	28.5	28.0	22.8	22.3
65 and older	16.5	10.5	16.0	18.2	18.0	15.4
Race						
White	74.9	67.4	69.3	68.4	70.8	70.1
Black or African American	4.3	7.2	6.0	23.6	19.4	10.9
Asian	6.3	12.6	18.9	2.3	0.6	7.2
Other	14.6	12.8	5.65	5.7	9.2	10.1
Hispanic ethnicity (% yes)	29.4	43.4	22.7	28.0	67.0	38.4
Household income						
Less than $25,000	22.9	21.1	14.2	21.5	29.2	22.6
$25,000 to $49,000	23.2	20.5	17.2	23.5	24.4	22.3
$50,000 to $74,999	16.2	15.6	15.6	17.2	15.8	16.1
$75,000 to $99,999	11.5	11.0	12.6	11.1	9.8	11.1
$100,000 to $149, 999	10.9	13.3	17.3	12.3	9.9	12.1
$150,000 or more	11.2	13.2	20.7	9.6	8.5	11.7
Educational attainment						
Less than high school	2.0	0.8	0.0	2.6	2.5	1.7
High school diploma or GED	33.8	35.2	8.6	33.7	39.1	33.1
Some college, technical school, or 2-year college degree	33.2	26.8	32.3	30.2	31.0	29.9
Four-year college degree	19.7	23.7	19.7	21.9	21.2	23.1
Advanced degree	11.3	13.5	11.3	11.6	10.9	12.2
One or more chronic health conditions (obesity, diabetes, heart disease, asthma, COPD, other lung diseases)	30.4	25.8	19.0	34.4	38.6	30.4
Pollen allergies	32.6	20.2	26.2	25.9	4.4	23.2
Disability (physical disability, deaf or hearing impaired, blind)	13.0	14.1	9.7	8.9	14.5	12.6
Mental health condition	12.0	8.1	4.5	14.6	9.6	10.5
Political party affiliation						
Republican	25.5	17.9	29.2	7.6	22.2	20.2
Independent, leans Republican	15.8	9.3	3.8	17.2	10.3	12.6
Democrat	39.0	51.4	35.3	57.5	40.7	45.3
Independent, leans Democrat	10.4	13.2	29.8	9.4	10.6	12.5
Prefers not to say	7.2	5.9	1.8	6.4	13.6	7.4
Extreme weather-related event within 50 miles of home in past 12 months^a^						
Extreme heat	88.6	72.2	84.4	46.0	41.1	69.1
Drought	56.8	58.6	59.4	9.7	13.6	42.5
Flooding	53.1	31.6	21.9	56.4	43.5	44.3
Wildfire	38.3	59.2	85.0	10.5	14.4	38.5
Hurricane	0.5	0.5	0.0	82.4	86.1	29.6
Tropical storm	9.0	12.7	4.9	78.6	82.3	33.4
Landslide or mudslide	5.0	26.8	21.7	1.6	3.4	11.0

^a^ Not mutually exclusive.

COPD, chronic obstructive pulmonary disease.

### Perceived importance of adaptation and mitigation approaches

Between 67.5% and 85.5% of respondents scored each approach greater than a midpoint rating of “3.” The mean ratings ranged from 3.90 (standard error [SE]: 0.06) to 4.43 (SE: 0.05) for adaptation approaches, and 3.91 (SE: 0.06) to 4.13 (SE: 0.06) for mitigation approaches (Table 2).

#### Health care

Controlling for health status, low income, and other covariates, Hispanic respondents had nearly twice the odds of emphasizing the need for improved access to hospitals and health systems for extreme weather-related health problems compared to non-Hispanic, non-Black respondents (odds ratio [OR]: 1.91, *p*=0.026) (Table 3).

**Table 3.** Perceived Importance of Public Health Approaches to Adaptation According to Social and Medical Factors

	Access to hospitals and health systems (*n*=544)	Require reinforcing construction and building repairs (*n*=542)	Emergency alert systems (*n*=511)	Services for those who can't afford to evacuate (*n*=545)	Evacuation transport for those without transportation (*n*=539)	Services for disabled/limited mobility/elderly (*n*=543)	Air conditioning units for low-income households (*n*=544)	Mental health services after extreme weather (*n*=544)
	OR (95% CI)	OR (95% CI)	OR (95% CI)	OR (95% CI)	OR (95% CI)	OR (95% CI)	OR (95% CI)	OR (95% CI)
Low income	1.12 (0.69–1.82)	**1.70 (1.03–2.81)**^a^	**1.76 (1.08–2.89)**^a^	0.96 (0.57–1.61)	1.01 (0.63–1.62)	—	**2.00 (1.24–3.22)**^b^	—
Poverty	—	—	—		—	**1.95 (1.02–3.75)**^a^	—	1.78 (0.99–3.19)
Race/ethnicity								
Black	1.93 (0.73–5.08)	—	1.75 (0.77–3.99)	**2.50 (1.19–5.23)**^b^	1.39 (0.61–3.21)	1.79 (0.89–3.62)	—	—
Hispanic	**1.91 (1.08–3.37)**^a^	—	**1.86 (1.05–3.30)**^a^	1.36 (0.75–2.45)	**1.88 (1.01–3.50)**^a^	1.54 (0.88–2.69)	—	—
Chronic conditions(s)	1.04 (0.64–1.71)	—	**1.69 (1.01–2.83)**^a^	—	—	—	—	—
Disability	1.95 (0.86–4.46)	—	—	**2.05 (1.02–4.14)**^a^	—	—	—	—
Mental health	1.06 (0.49–2.30)	—	—			—		**1.86 (1.00–3.43)**^a^
Age group	1.03 (0.91–1.15)	1.03 (0.93–1.14)	1.10 (0.98–1.24)	**1.12 (1.00–1.26)**^a^	**1.17 (1.04–1.33)**^a^	**1.13 (1.01–1.26)**^a^	**0.88 (0.95–2.59)**^a^	**0.87 (0.78–0.97)**^a^
Political^d^	1.55 (0.98–2.46)	**1.76 (1.06–2.93)**^a^	0.70 (0.43–1.13)	1.63 (0.98–2.71)	**2.43 (1.52–3.88)**^c^	1.12 (0.71–1.77)	1.57 (0.95–2.59)	1.18 (0.75–1.86)
County^e^								
Los Angeles	1.69 (0.95–3.02)	**1.99 (1.02–3.89)**^a^	1.41 (0.77–2.58)	1.11 (0.55–2.24)	1.66 (0.91–3.05)	1.06 (0.59–1.90)	0.92 (0.48–1.77)	1.12 (0.65–1.94)
Orange (CA)	0.86 (0.36–2.06)	1.26 (0.46–3.48)	0.83 (0.40–1.71)	0.46 (0.17–1.29)	0.84 (0.31–2.25)	0.46 (0.17–1.26)	0.50 (0.18–1.38)	0.65 (0.24–1.75)
Broward	1.49 (0.76–2.92)	1.61 (0.79–3.28)	0.95 (0.50–1.81)	0.73 (0.41–1.30)	1.04 (0.56–1.94)	0.71 (0.36–1.41)	0.90 (0.47–1.73)	1.15 (0.64–2.07)
Miami-Dade	1.23 (0.56–2.71)	1.74 (0.90–3.38)	0.72 (0.32–1.64)	1.24 (0.56–2.73)	1.69 (0.73–3.90)	0.91 (0.44–1.88)	1.04 (0.53–2.07)	1.32 (0.72–2.42)
	Wald χ^2^=22.49, df: 12, *p*=0.032	Wald χ^2^=24.22, df: 7, *p*=0.001	Wald χ^2^=24.25, df: 10, *p*=0.007	Wald χ^2^=24.01, df: 10, *p*=0.008	Wald χ^2^=27.11, df: 9, *p*=0.001	Wald χ^2^=16.67, df: 9, *p*=0.054	Wald χ^2^=22.00, df: 7, *p*=0.003	Wald χ^2^=19.82, df: 8, *p*=0.011

Boldface indicates statistical significance (^a^*p*<0.05, ^b^*p*<0.01, ^c^*p*<0.001) using multivariable ordered logistic regression.

^d^ Ref.: Republican (including Republican-leaning Independents).

^e^ Ref.: Maricopa County, AZ.

CI, confidence interval; OR, odds ratio.

#### Infrastructure improvements

Low-income respondents had significantly higher odds of viewing requirements that landlords and building owners do construction or repairs to make housing safer during extreme weather with high importance, compared to higher-income respondents (OR: 1.70, *p*=0.037), controlling for other covariates.

#### Support services

Respondents in low-income households had twice the odds of stressing the importance of providing air conditioning units to people who cannot afford them (OR: 2.00, *p*=0.005), compared to those in higher-income households, controlling for other covariates (Table 3). Black respondents had significantly greater odds of seeing the improvement or creation of services to help people who can't afford to evacuate in extreme weather as being highly important compared to non-Hispanic respondents of other races (OR: 2.50, *p*=0.015), controlling for disability and other covariates. Disabled respondents were twice as likely to find the improvement or creation of these services of high importance (OR: 2.05, *p*=0.044), controlling for race/ethnicity and other covariates. Hispanic respondents stressed the importance of transportation services for those without access to transportation, controlling for other covariates (OR: 1.88, *p*=0.047). Respondents who lived in households below the federal poverty level had nearly twice the odds of perceiving the improvement or creation of services to help people with limited mobility in extreme weather events (OR: 1.95, *p*=0.044) as being of high importance, controlling for race/ethnicity and other covariates. Respondents with a mental health condition were more likely to stress the importance of providing mental health services after extreme weather (OR: 1.86, *p*=0.047), controlling for household poverty and other covariates.

#### Communication

Controlling for other covariates, Black respondents were over three times more likely to stress the importance of receiving information on how communities can prevent extreme weather from becoming stronger or more frequent in the future (OR: 3.44, *p*=0.001) compared to non-Hispanic respondents of other races; Hispanic respondents had nearly twice the odds of seeing this as important (OR: 1.93, *p*=0.028) (Table 4). Compared to other respondents, Black and Hispanic respondents had more than twice the odds of believing in the importance on receiving yearly updates about how extreme weather is changing over time (OR: 2.24, *p*=0.024 and OR: 2.18, *p*=0.010, respectively). Controlling for race/ethnicity, chronic conditions, and other covariates, low-income households were significantly more likely to perceive the improvement of emergency alert systems as being highly important compared to higher-income households (OR: 1.76, *p*=0.024), as were Hispanic respondents (OR: 1.86, *p*=0.034) and respondents with chronic conditions (OR: 1.69, *p*=0.044) (Table 3).

**Table 4.** Perceived Importance of Public Health Approaches to Mitigation According to Social and Medical Factors

	Communication about local mitigation actions (*n*=544)	Communication about changes in extreme weather (*n*=540)	Regulation of private sector GHG emissions (*n*=541)	Regulation of household GHG emissions (*n*=543)	“Cooling” design features in public buildings (*n*=546)	Financial incentives for “cool” roofing (*n*=543)	Subsidized costs of energy-efficient appliances (*n*=542)	Intergovernmental cooperation on mitigation (*n*=541)
	OR (95% CI)	OR (95% CI)	OR (95% CI)	OR (95% CI)	OR (95% CI)	OR (95% CI)	OR (95% CI)	OR (95% CI)
Low income	0.94 (0.58–1.52)	1.22 (0.77–1.93)	**1.64 (1.04–2.59)**^a^	—	**1.61 (1.00–2.57)**^a^	**1.68 (1.04–2.74)**^a^	**1.99 (1.28–3.09)**^b^	1.08 (0.67–1.77)
Poverty	—	—	—	**2.22 (1.30–3.81)**^b^	—	—	—	—
Race/ethnicity								
Black	**3.44 (1.63–7.25)**^c^	**2.24 (1.11–4.49)**^a^	1.57 (0.74–3.36)	1.61 (0.76–3.38)	—	1.10 (0.52–2.34)	**2.85 (1.19–6.84)**^a^	1.79 (0.90–3.57)
Hispanic	**1.93 (1.07–3.48)**^a^	**2.18 (1.20–3.96)**^a^	1.73 (1.00–2.98)	**2.12 (1.26–3.55)**^b^	—	1.64 (0.84–3.19)	**2.14 (1.25–3.69)**^b^	**2.32 (1.33–4.05)**^b^
Age group	1.06 (0.94–1.20)	1.05 (0.93–1.18)	1.05 (0.93–1.18)	1.02 (0.90–1.15)	1.02 (0.91–1.13)	1.01 (0.90–1.14)	0.97 (0.86–1.08)	1.00 (0.90–1.13)
Political^d^	**2.25 (1.40–3.61)**^c^	**1.64 (1.04–2.58)**^a^	**2.20 (1.41–3.44)**^c^	**2.40 (1.53–3.79)**^c^	**1.68 (1.07–2.63)**^a^	2.08 (1.28–3.38)^b^	1.12 (0.72–1.75)	**1.94 (1.20–3.16)**^b^
County^e^								
Los Angeles	0.87 (0.46–1.65)	1.08 (0.59–1.96)	1.28 (0.74–2.21)	1.48 (0.86–2.56)	1.01 (0.58–1.76)	1.69 (0.90–3.17)	1.08 (0.59–1.97)	1.68 (0.91–3.44)
Orange (CA)	0.47 (0.21–1.06)	0.54 (0.23–1.30)	0.55 (0.23–1.33)	1.10 (0.37–3.28)	0.59 (0.22–1.54)	0.80 (0.33–1.95)	0.45 (0.16–1.30)	0.90 (0.40–2.02)
Broward	0.99 (0.53–1.83)	0.74 (0.38–1.43)	0.72 (0.39–1.35)	1.17 (0.63–2.16)	0.83 (0.43–1.58)	0.90 (0.43–1.88)	0.79 (0.45–1.40)	1.17 (0.66–2.08)
Miami-Dade	1.03 (0.48–2.23)	0.98 (0.48–1.98)	0.66 (0.30–1.41)	0.92 (0.49–1.70)	1.40 (0.71–2.77)	1.16 (0.51–2.63)	0.84 (0.42–1.65)	1.03 (0.48–2.30)
	Wald χ^2^=33.99, df: 9, *p*<0.001	Wald χ^2^=22.52, df: 9, *p*=0.007	Wald χ^2^=30.14, df: 9, *p*<0.001	Wald χ^2^=43.12, df: 9, *p*<0.001	Wald χ^2^=14.38, df: 7, *p*=0.045	Wald χ^2^=33.40, df: 9, *p*<0.001	Wald χ^2^=31.95, df: 9, *p*<0.001	Wald χ^2^=25.14, df: 9 *p*=0.003
	Community gardens and local agriculture (*n*=542)							
	OR (95% CI)							
Low income	**1.66 (1.05–2.63)**^a^							
Race/ethnicity								
Black	1.33 (0.63–2.80)							
Hispanic	1.59 (0.93–2.71)							
Age group	0.95 (0.84–1.06)							
Political^d^	1.41 (0.88–2.26)							
County^e^								
Los Angeles	1.51 (0.85–2.68)							
Orange (CA)	0.57 (0.21–1.53)							
Broward	1.21 (0.65–2.26)							
Miami-Dade	0.93 (0.49–1.77)							
	Wald χ^2^=20.52, df: 9, *p*=0.015							

Boldface indicates statistical significance (^a^*p*<0.05, ^b^*p*<0.01, ^c^*p*<0.001) using multivariable ordered logistic regression.

^d^ Ref.: Republican (including Republican-leaning Independents).

^e^ Ref.: Maricopa County, AZ.

GHG, greenhouse gas.

#### Structural approaches

Respondents from low-income households were significantly more likely to place high importance on requirements that public buildings and spaces be designed to make cities cooler compared to higher-income households (OR: 1.61, *p*=0.049) (Table 4). Those from low-income households also had greater odds of stressing the importance of laws that require businesses to lower local pollution and greenhouse gas emissions compared to higher-income households (OR: 1.64, *p*=0.033), controlling for race/ethnicity and other covariates. Respondents from households below the federal poverty level had over twice the odds of placing high importance on laws that required households to lower local pollution and greenhouse gas emissions, compared to households above the poverty level and controlling for race/ethnicity and other covariates (OR: 2.22, *p*=0.004); Hispanic respondents expressed greater perceived importance of these laws compared to non-Hispanic, non-Black respondents (OR: 2.12, *p*=0.005) controlling for poverty and covariates.

Controlling for race/ethnicity and other covariates, low-income households had greater odds of believing in the importance of loans, tax breaks, rebates, or giveaways to encourage residents to install cool roofing (OR: 1.68, *p*=0.036) compared to higher-income households (Table 4). Low-income households similarly had higher odds of placing greater importance in help with covering the costs of energy-efficient appliances (OR: 1.99, *p*=0.002), controlling for race/ethnicity and other covariates, as did Black (OR: 2.85, *p*=0.019) and Hispanic (OR: 2.14, *p*=0.006) respondents compared to respondents of other race/ethnicities.

#### Intergovernmental policies

Controlling for low income and other covariates, Hispanic respondents were more than twice as likely as non-Hispanic, non-Black respondents to stress the importance of intergovernmental cooperation to prevent extreme weather from becoming worse (OR: 2.32, *p*=0.001) (Table 4).

#### Approaches with co-benefits

Respondents from low-income households had greater odds of stressing the importance of supporting and expanding community gardens and local agriculture compared to higher-income respondents (OR: 1.66, *p*=0.030), controlling for race/ethnicity and other covariates (Table 4).

## Discussion

Residents of regions currently being impacted by climate change emphasize the importance of adaptation and mitigation approaches. The three regions sampled for this study had been affected by costly and dangerous extreme weather and related hazards in the previous year (i.e., hurricanes, wildfires, extreme heat).^22–24^ Adaptation measures that bolster emergency response activities in acute situations were among the most critical issues that respondents felt local governments could do to protect health and safety during extreme weather and environmental hazards.

This study reveals that socially and medically vulnerable community members perceive certain interventions to be especially important, and significantly more so compared to less-vulnerable residents. Individuals from low-income households expressed stronger support for community-level structural policies compared to those with higher income. The structural interventions emphasized by low-income residents mainly address mitigation (laws on households and businesses to lower local pollution and greenhouse gas emissions; subsidizing the costs of energy-efficient appliances to low-income residents; providing financial incentives to homeowners to install cool roofs) with some adaptation benefits (requiring public spaces to implement heat island reduction strategies such as green or cool roofs, planting vegetation, installing cool pavements). Those from low-income households also expressed greater interest in the expansion of community gardens and local agriculture than higher-income community members, a mitigation approach that can provides co-benefits for public health by improving access to fresh fruits and vegetables. Low-income neighborhoods frequently have more limited access to affordable and fresh food, contributing to higher rates of obesity and related chronic conditions.^21^

This study provides evidence that racial and ethnic groups at increased risk of health impacts from climate change desire greater communication from their local government about acute and long-term changes in extreme weather, and mitigation. Black or Hispanic respondents felt more strongly that receiving information on preventing increases in intensity and frequency of extreme weather in the future was important, and were significantly more likely to emphasize the importance of yearly updates about local changes in extreme weather over time, compared to non-Black, non-Hispanic respondents. Hispanic respondents were more likely than non-Hispanics to emphasize the importance of making improvements in emergency alert systems during extreme weather events.

Race/ethnicity was also associated with perceiving the importance of aid during extreme weather. Hispanic respondents called for improved access to health care during extreme weather events more so than non-Hispanics. Black respondents stressed the importance of providing services to people who cannot afford to evacuate in extreme weather. Hispanic respondents had significantly greater interest in transportation to facilitate evacuation in extreme weather for those who could not afford it.

Certain medical vulnerabilities were associated a greater interest in services in extreme weather situations. Those with physical disabilities emphasized the importance of services that help those with financial barriers to evacuation, and respondents with mental health conditions were more likely to stress the importance of mental health services after extreme weather events. Having a chronic illness was associated with greater perceived importance of improved emergency alert systems, but otherwise no systematic differences were found.

### Limitations

This study focuses on within-sample differences in attitudes about the importance of interventions according to respondent vulnerabilities to extreme weather. The prevalence of attitudes expressed by this sample should be interpreted with caution due to the nonprobability sampling technique used. Post-stratification weights, combined with sampling quotas, helped to achieve a sample that closely resembles the demographic characteristics of the counties of interest, but it cannot be considered representative. Online panels are vulnerable to selection bias, as those enrolling in a panel may systematically differ from the general population. Despite these limitations, an online panel provided unique benefits for this study. Achieving a probability-based sample within this study's restricted geographic areas would be costly and vulnerable to low response rates. A short data collection period helped to prevent history bias if new extreme weather events occurred during the study.

### Health equity implications

Community members with greater vulnerability to the health impacts of climate change indicate elevated levels of interest in preparedness for, and prevention of, its impacts. Low-income residents in regions affected by extreme weather are among the most supportive of local structural policies toward mitigation, and interventions that offer public health co-benefits. Strengthening social services during extreme weather events is also critically important to low-income residents. Further research can identify barriers and contribute to the development and refinement of culturally competent, accessible interventions that facilitate decisions surrounding preparedness and evacuation during extreme weather.

Based on the interests expressed in this study, greater communication by local governments with Black and Hispanic constituents about climate change processes and mitigation is recommended. Emergency and risk communication should also better target Hispanic community members during acute events, as Hispanic respondents to this survey appeared to have more limited access to both emergency alerts and health care services for health problems due to extreme weather and environmental hazards. Such outreach should consider linguistic, cultural, or dissemination barriers that residents encounter during such events.

Determining the needs and priorities of residents is a localized process that involves the engagement of diverse groups of community members. Local jurisdictions should take care to ensure that adaptation and mitigation approaches address the expressed concerns of community members most affected by increasingly extreme weather and other environmental hazards, as they may differ from constituents overall.

## Abbreviations Used

OR: odds ratio
SE: standard error
